# Supplementary material for: The supply is there. So why can’t pregnant and breastfeeding women in rural India get the COVID-19 vaccine?
Source: PLOS Glob Public Health. 2022 Dec 6;2(12):e0001321. doi: 10.1371/journal.pgph.0001321 (PMC10022347; doi:10.1371/journal.pgph.0001321)
Supplement: S1 Text — (DOCX) [file pgph.0001321.s001.docx]

Inclusivity in global research

PLOS’ policy on inclusivity in global research aims to improve transparency in the reporting of research performed outside of researchers’ own country or community and ensures that PLOS publications reporting global research adhere to high standards for research ethics and authorship. Authors of relevant research articles may be asked to complete the questionnaire below, which outlines ethical, cultural, and scientific considerations specific to inclusivity in global research. This questionnaire may be requested when researchers have travelled to a different country to conduct research, if research uses samples collected in another country, research with Indigenous populations or their lands, or if research is on cultural artefacts. Researchers travelling to another country solely to use laboratory equipment will not normally be required to complete the questionnaire. However, the questionnaire can be requested at the journal’s discretion for any submission – if you have been requested to complete this questionnaire by the PLOS journal you submitted to, please do so.

Please complete the questionnaire below and include this as a Supporting Information file with your manuscript. Note that if your paper is accepted for publication, this checklist will be published with your article in the supporting information files. Please ensure that you reference the checklist in the main body of your manuscript. We suggest adding a subsection ‘Inclusivity in global research’ to your Methods section and adding the following sentence: “Additional information regarding the ethical, cultural, and scientific considerations specific to inclusivity in global research is included in the Supporting Information (SX Checklist)”

The questions have been designed to be applicable to a wide range of study types, and there are subsections for both human subjects research and non-human subjects research. If any of the questions are not relevant to your research please mark them as “N/A” as appropriate.

**Ethical considerations, permits and authorship**

*This section is applicable to all research types.*

Provide details as to who granted permissions and/or consent for the study to take place in the Methods section of your manuscript. This should include the names of **all** ethics boards, governmental organizations, community leaders or other bodies that provided approval for the study. If individuals provided approval refer to these people by their role or title but do not list their name(s).

Reported on page number: 9

If there were any deviations from the study protocol after approval was obtained please provide details of these changes in the Methods section of your manuscript.
Did this study involve local collaborators that are residents of the country where the research was conducted or members of the community studied? If you do not have any authors from said communities, please provide an explanation for this below.

Reported on page number: NA

The following co-authors are from the country where the research was conducted, including from local NGOs and academic institutions.

Preetika Sharma, PhD

Mona Duggal, MD, MHS

Navneet Gill, PhD

Jagriti Gupta, MBA

Vijay Kumar, MD

Jasmeet Kaur, M.Tech

Pushpendra Singh, PhD

Everyone listed as an author should meet PLOS’ criteria for authorship and all individuals who meet these criteria should be included in the author byline, rather than the acknowledgements. Authorship criteria is based on the International Committee of Medical Journal Editors (ICMJE) Uniform Requirements for Manuscripts Submitted to Biomedical Journals - for further information please see here: <https://journals.plos.org/plosone/s/authorship>.

**Human subjects research (e.g. health research, medical research, cross-cultural psychology)**

Did you obtain written informed consent from a representative of the local community or region before the research took place? How did you establish who speaks for the community? Details of written informed consent obtained from study participants should be reported separately in the Methods section of your manuscript.

How did members of the local community provide input on the aims of the research investigation, its methodology, and its anticipated outcome(s)?

When engaging with the local community, how did you ensure that the informed consent documents and other materials could be understood by local stakeholders?

Since 2013, SWACH has been working in collaboration with NHM Haryana in two districts Viz Ambala & Yamunanagar. ASHAs deployed by the government report from all villages in these two districts all pregnancy and child birth on a daily basis to SWACH by phone. The information obtained is recorded on computers to document key indicators related to pregnancy, child birth & events during postnatal period. This has been done as a collaborative effort to strengthen home based postnatal care. An M.O.U has been signed for sustenance of the efforts.

On receipt of information from ASHAs SWACH supervisor establish contact with families to obtain further information & provide guidance based on needs.

Since December 2018 SWACH has formed what are App. groups for pregnant women & for families having children 0-3 years of age. These groups have been divided further into subgroups (0-6 months, 6-12 months, more than 1 year, children for low birth weight and children with birth defects).  SWACH staff established telephone contact to obtain consent from the interested families. Right at the outset they were given the option to exit the group whenever they wished.

 The groups change as per the age and stage. When we started the R & R project there were about 2400 members in 12 different groups. In the What’s app groups, there is a daily exchange of information, articulation of problems and concerns, thematic discussion related to key health promoting interventions. Audios of 4-10 minutes duration are posted in the group and advice regarding telephone conversation. Families share videos of their children that are reviewed and comments posted for guidance. With the passage of time members of the groups have extended beyond the geographical boundaries of the two districts. Therefore, it is fair to state that the membership in the different groups was not limited to these two districts.

In the context of the R & R project the survey was carried out by digital means and appropriate approvals obtained as per consensus of the partners. Appropriate information in local language was shared from time to time on whats app walls and consent of the participants was obtained digitally. A similar approach was also used while conducting in depth interviews.

1.    Did you obtain written informed consent from a representative of the local community or region before the research took place? How did you establish who speaks for the community?

As described above, it is not possible to identify any local or regional leadership for any consultation or approval. It should be pointed out that no leader could be identified during the 3 years of operation of these groups. Hence we relied on the feedback of our clients.

2.    How did members of the local community provide input on the aims of the research investigation, its methodology, and its anticipated outcome(s)?

Before starting the project and during the course of the project relevant information was provided to inform about the aims of the proposed project and its details in the form of text messaging on the walls in local language. Whenever necessary, videos were posted to guide the clients.  In the chats clients were encouraged to share their experiences with BOTs and also write the difficulties they faced and suggestions for its improvement. In summary, a problem solving approach was used throughout.

3. When engaging with the local community, how did you ensure that the informed consent documents and other materials could be understood by local      stakeholders?

Every local stakeholder was informed about the study, its objective and consent through information on the wall. They had the option to either enroll or not participate .Enrollment was totally voluntary.

Will the findings of the research be made available in an understandable format to stakeholders in the community where the study was conducted (e.g. via a presentation, summary report, copies of publications, etc.)? Please provide details of how this will be achieved.

So far we have not done this because there can be a conflict if the findings of research are shared prior to publication. We agree that important summary findings and experiences should be shared with the stakeholders in an easy to use format .Once we have access to key findings & conclusions we will make it available to the stakeholders on the various platforms we are using viz whatsapp group, twitter, zoom meetings etc

**Non-human subjects research using specimens/ animals collected as part of the study, or those housed in archival collections. Examples include archaeology, paleontology, botany and zoology.**

Did the permission you obtained from a local authority to perform the study include an agreement on access to outputs and benefit sharing? This may include procedures to enable fair distribution of the benefits and resources arising from the research performed. Please include any details of Prior Informed Consent and Benefit Sharing Agreements obtained. These may be required by field-specific regulations, for example the Convention on Biological Diversity (CBD) and the associated Nagoya Protocol.

If the material used in your study was imported, please A) provide the year it was imported and B) indicate whether permits were obtained to import/export the materials used, C) provide details of any permits obtained. If this information is not available, please indicate this.

If you used archival specimens, please state how the material used in your study was acquired by the institute it is held in and provide details of any permits obtained for the original excavations/ sample collection. If this information is not available, please indicate this.

How was the potential cultural significance of the materials collected in your study to local communities considered in your research design? Were Indigenous peoples and/or local researchers and institutions involved with archaeological excavations / collection of specimens? If so, please provide a description of their involvement.

If your manuscript includes photographs of human remains please indicate whether authors obtained permission from descendants or affiliated cultural communities to do so.
