# Supplementary material for: The supply is there. So why can’t pregnant and breastfeeding women in rural India get the COVID-19 vaccine?
Source: PLOS Glob Public Health. 2022 Dec 6;2(12):e0001321. doi: 10.1371/journal.pgph.0001321 (PMC10022347; doi:10.1371/journal.pgph.0001321)
Supplement: Attachment — Submitted filename: PlosGlobalHealth response to reviewers.docx [file pgph.0001321.s002.docx]

To the editors and reviewers

First, thank you so much for these helpful and detailed comments. Second, we are so sorry that the correspondence from you went to the lead author’s spam folder and thus we were so delayed in getting these comments back to you. In the intervening time, we had already decided to add more depth to our analysis and included multivariate models looking at factors associated with vaccine uptake, to make the paper more than descriptive. We hope that you will agree that these add depth to the previous version of the paper.

Below we have responded to each of your comments point by point. We are very excited about this paper and grateful for your comments which have greatly strengthened it!

Thank you,

Nadia Diamond-Smith

Reviewer #1: Thank you for an opportunity to review this article, which attempts to address a very important topic of low uptake of the COVID-19 Vaccine among pregnant and lactating women in one rural setting in India. The authors seem to have made a rigorous attempt to capture data through mixed methods, but much of the details needed to interpret the paper are missing from the paper at present. I would suggest a revised paper with more contextual details, methodological nuances such as details of the sampling size and strategy, detailed reporting of quantitative tables (there are no tables) and qualitative findings (few scattered themes reported), and a deeper discussion section in relation to current literature.

Thank you for these helpful comments.

More details are below

Introduction

Important dates, COVID- context in India particularly of vaccination, type of vaccines available, the CoWin App and digitalization -perhaps a timeline here? when did vaccination begin, when did it begin for pregnant women (highlighting the fact that it was not approved for pregnant women initially)

We have added a figure which hopefully will help situate the reader as to the situation leading up to this study in India.

-a strong argument for why this study was done. What does it add to what we already know in literature?
- description of the context is important, currently there is no description of the study setting. If the state name/district was masked, perhaps how does this limit interpretations of the data. For instance, study (10) has shown that 93% of women reported intentions to get vaccinated. Was something different in this context?

We looked back at that paper, and it unfortunately did not mention where in India the data was collected (although we think it was national because online survey). However, we have added the following, and also, we added a regression to look at factors associated with uptake as a way of adding to the contribution of this paper.

“A 2021 systematic review of studies looking at vaccine uptake in pregnant women found that the biggest predictors of vaccine uptake trust in the effectiveness and safety of the vaccine, communication about safety of the vaccine in pregnancy, trust in public health agencies, anxiety about COVID-19 and other COVID-19 safety behaviors (11). Age, education and socio-economic status were also associated with vaccination in pregnancy women. However, this review mostly included papers with data from high income countries, with only one paper from 2020 having collected data in India. While there are a number of papers from across India looking at vaccine acceptance and hesitancy (2,12–15), only one focuses on pregnant and postpartum women. The 2022 study from northern India explored barriers to vaccination among pregnant and postpartum women, collecting data from a mostly urban, well-educated and highly vaccinated population. This study found large differences by socio-economic and rural/urban status, and found that concerns over the impacts on the baby and lack of inclusion of women in vaccine safety trials were main contributors to vaccine hesitancy (delay in acceptance or refusal of vaccination despite availability) (16). More, mixed-methods research is needed with rural, less well off, and un-vaccinated groups of pregnant and postpartum women to understand contributors and barriers to vaccine uptake. With this in mind, we hypothesized that a variety of factors would influence vaccine uptake among pregnant and recently delivered women in India. Specifically, belief in the effectiveness and safety, communication from trusted health care providers, norms around community acceptance/uptake, and socio-economic factors.”

Methods

Detailed description of survey methods- where was it done, sample size calculations, non response rates, survey tool and its dimensions ( might help to attach), data cleaning, analysis, etc Details of qualitative methods- purposive sample, saturation, non-response, key themes that were asked, interview guide used for facilitation. Transcription, analyses

We have greatly expanded the methods, see below:

“The surveys were conducted telephonically. The participants were active members of WhatsApp groups run by a local NGO that was a collaborator on the project. A list of 552 eligible women, from a sample oof about 5,000, was provided to the research assistants. Women who were either pregnant or had delivered within 1 year were eligible for the survey. The list included their name, mobile and date of delivery. These women were called one by one down the list provided by the research assistant. Women were read an informed consent and asked to provide verbal consent. A survey call was scheduled based on time convenient for the women. Most of the surveys were completed in one call and few were done in parts based on the availability of the participant. Out of about 450 women called, 300 complete surveys were taken. Some women did not pick the call, or only completed half of the survey. The team began to take the surveys in the first week of November, 2021 and 300 surveys were completed by Nov 27, 2021. The women were called to again to get the missing information wherever possible.

The survey tool was developed and finalized by the team members and was put on web application RedCap. It included questions on vaccine acceptance, barrier, hesitancy and socio-demographics. The redcap data was relooked to fix any incorrect data or missed areas. Quantitative survey data was analyzed using descriptive statistics (frequencies, percentages) using STATA version 15.

At the end of the telephonic survey, women were asked if they would be willing to be contacted to participate in longer follow-up in-depth interviews.  Later, the research associate reached out to those who agreed for a telephonic in-depth interviews. of the 27 called, 15 women agreed to give an interview. The interviews were conducted till the saturation in the data was achieved. In depth interviews that lasted from 35-70 minutes. In depth interviews were collected until data saturation was reached, which was determined by the study team conducting the interviews. Some surveys and interviews were taken on multiple calls due to the time constraints of the participants. The interviews were conducted in Hindi. They were later transcribed and translated into English. A codebook based on the key themes covered in the interview was made to analyse the interviews. These were then analysed by the three members from the research team using dedoose software. In the context of covid-19 vaccine, key themes covered in the interview included general impact of covid-19 pandemic, its impact on their pregnancy, their own thoughts on covid-19 vaccine, thoughts of their family members, views of community and perinatal care provider on covid-19 vaccine, decision making, sources of information and understanding of bot technology.

We also added the following about the site “The study was carried out in lower and upper middle class populations. The population resides in rural and suburban areas in two districts. Our estimates are that about 60 % of the respondents were from two districts in north India. In terms of health and socio demographic characteristics, these districts rank in the middle during the past decade. In total, we conducted 300 phone surveys and 15 in-depth interviews with rural women. The eligibility criteria were to include pregnant and recently delivered women who were breastfeeding (up to 6 months postpartum). Since this was a formative, descriptive study, we estimated that 300 respondents would be sufficient to describe trends in vaccination, barriers and hesitation.”

Details of the qualitative and quantitative demographics of participants needed.

Added in a table.

Findings

It might make sense to separate the qualitative and quantitative findings and present them separately.

Thank you for this comment. We discussed this as a team and feel strongly that the qual and quant should be presented together to really take advantage of the mixed methods nature of this data. However, we have organized the results better to hopefully make it easier for the reader to follow our findings.

In the survey section of the findings, please report main data tables-example- intention to get vaccine,sources of information, trust, etc. also if the responses differed by age, family type, parity, the trimester they are in, other socio demographics. We don’t have much details of the 500 people surveyed in the findings. Discuss the key tables in details.

We have added tables with the data.

In the qualitative section, one can pull from the data table and try to explain some of the ‘whys’ of the findings. But this needs to be done in 3-5 well-defined themes, for right now there is a long write-up that is a bit difficult to read. For instance, lines 140 -152 have several themes jumbled up- reasons for not getting vaccinated, some idea of trust and on uptake, etc. Quotes need to be reported in lines with how qualitative data is usually reported (in double quotes, with some description of the participant demographics)- so that quotes can be interpreted by the reader with ease. More direct quotes can be included to give readers a better flavour of the field. One can also use tables to present summarized qualitative data or data from a few cases that highlight important issues. The richness of the interviews need to come out in this section

We have added some more quotes and also the age and status (breastfeeding or pregnant) of each respondent. We didn’t add more detail since that was not the focus of this paper (for example, we are not focused on differences by participant characteristics). We have reorganized the results, and added headers to try to make our main themes and points clearer and more organized.

Discussion

-the limitations of the study need to be discussed. What were some of the limitations of doing phone interviews? How was non response dealt with? Do we leave out specific sections of the population ( the most vulnerable in fact) if we do phone interviews?

We have added the following:

“Additionally, all women had phones, and thus, while a large proportion noted having ration cards (a marker of poverty), this sample did not represent the least well-off populations in this setting (those without access to phones). Collecting data over the phone also posed limitations including (a) challenges in finding a suitability time for interview and potential lack of privacy for the respondents (b) Clarity of communication due to echo and voice cracking and (c) having to break up interviews over multiple phone calls for some participants”

-lines 209-211 seem particularly important to me, based on what I know of the Indian context. But this point hasn’t been detailed in the findings section anywhere.

We have tried to expand upon this section (we think you are referring to provider refusal), see additional comments above.

-what has been found from other contexts on pregnancy and vaccination. There is a lot of literature in the non-covid areas as well on this.

Interesting point, we have added this “A study from India among pregnant women and their health care providers regarding influenza vaccinations also found that health care providers were the most trusted sources for advice on the vaccination, and also that high interest among women was high, there was more uncertainty among their health care providers—although it did not seem as extreme as in this case. Perhaps the novelty of the COVID-19 vaccine made providers additionally hesitant. “

- What is particularly different about India. In fact in india, pregnant women are offered vaccines ( TT) regularly, what is it that made them hesitate with regard to this vaccine? Vaccination was also happening in the private sector- in this context, did the women have access to this option.

Re: COVID compared to other vaccines: The different part was about the CORONA virus and not about the vaccines in general. While the Indian population is generally well-aware of vaccines and participates in it, however, these vaccines have been part of their lives for multiple decades and there is neither an apprehension of the disease nor of the vaccine thanks to years of awareness campaigns and practice. On the other hand, the COVID-19 was a very new virus and myths were associated with it. The media reports also exaggerated the risks associated with the virus and vaccines. Similarly, the short-time in which the vaccines were developed  created hesitation. Unlike other vaccines, which were time-tested and for well-known diseases, the COVID-19 vaccines came suddenly and that possibly created anxiety. We have added a comment related to this issue in the discussion “Perhaps the novelty of the COVID-19 vaccine made providers additionally hesitant; the same could be true for hesitancy among community members as well, who are used to and accepting of other vaccines in pregnancy, such as tentanus.

Re: Private sector: Yes. Vaccination was accessible to the private sector throughout the course of the study while the vaccine was provided free of cost in government facilities. However, the private sector option was paid and costlier and they also only operated through pre-established entities, e.g. their hospital premises. The government provided the same vaccines free of cost and also set-up vaccination drives and centers in remote areas to reach everyone.

We have added “In this setting, the vaccine was available free of cost in government facilites, and for a charge in private facilities.”

-line 220- if the main reason for being unable to get a covid vaccine was due to the provider hesitancy, then should we not be educating providers rather than women as being suggested here. perhaps one needs to look at the main 3-5 reasons for low uptake, and see what specific measures (long-term and short-term) can be taken to address these. A table here would help to summarize these measures.

We have clarified that this was a primary contributor, added a table with main barriers, and have added to the discussion. “Healthcare provider refusal to vaccinate was a primary contributor to women in our sample being unable to receive a COVID-19 vaccine. This finding came out strongly in the qualitative and quantitative data—women wanted the vaccine while pregnant but were be advised not to get it, or were outright refused the vaccine. It is clear that education to providers about the safety of the vaccine in pregnancy was a missed opportunity and led to many women remaining unvaccinated in pregnancy, despite a desire for the contrary. This education would need to target all levels of health care providers, from specialized perinatal care providers to community health workers, and likely be ongoing and multi-pronged. Additionally, on a broader level, helping providers think through balancing the reality that pregnancy is a time of higher risk in some ways with the fact that COVID in pregnancy posed many risks could have helped shift the paradigm.”

Apologies again for the long list of suggestions. I feel that such topics are important to work with. It is just that the manuscript at present needs more work in order for it to convey the rigor of the methods used as well as the depths of the findings. My very best wishes for the revisions.

Reviewer #2: Dear authors,

This is an important, timely paper, and I commend you for conducting this research. I would like to make some suggestions that will tighten the arguments.

Introduction: 
- Lines 73-76: Be sure to specify that in a reversal of what was witnessed worldwide, where men had higher Covid-19 fatality rates than women, in India women had a higher fatality rate

Great point, We have added the following “In India gender has played an important role in structuring how the COVID-19 pandemic has impacted health outcomes with women bearing an unequal COVID-19 mortality compared to men, including among younger women of reproductive age (4,5). This is contrast to trends globally, where men had higher mortality than women.”

- Lines 83-87: The first and second part of the sentence have little in common except temporality - perhaps flip the two parts

Thank you, we have edited these sentences to have the transition and main point clearer. “Unvaccinated pregnant women are at even more risk of having adverse birth outcomes (8). COVID-19 contributed greatly to maternal mortality as well. Shortly before the Indian government issued guidance that the COVID-19 vaccine was approved for pregnant and breastfeeding women in India (July 2^nd^, 2021), Mumbai, the second most populous city in India, declared that COVID-19 was the leading cause of death among new mothers between 2020-2021 (9).”

Methods:
- Please provide an overview of topics covered in the phone survey and in-depth interview questionnaire 
- What % of women from the phone survey agreed to a follow-up interview? What % of these women were eventually interviewed? Did those who agreed to be interviewed differ in any way from those who did not? (just on some basic demographic measures)

We have greatly expanded the methods, see below:

“The surveys were conducted telephonically. The participants were active members of WhatsApp groups run by a local NGO that was a collaborator on the project. A list of 552 eligible women, from a sample oof about 5,000, was provided to the research assistants. Women who were either pregnant or had delivered within 1 year were eligible for the survey. The list included their name, mobile and date of delivery. These women were called one by one down the list provided by the research assistant. Women were read an informed consent and asked to provide verbal consent. A survey call was scheduled based on time convenient for the women. Most of the surveys were completed in one call and few were done in parts based on the availability of the participant. Out of about 450 women called, 300 complete surveys were taken. Some women did not pick the call, or only completed half of the survey. The team began to take the surveys in the first week of November, 2021 and 300 surveys were completed by Nov 27, 2021. The women were called to again to get the missing information wherever possible.

The survey tool was developed and finalized by the team members and was put on web application RedCap. It included questions on vaccine acceptance, barrier, hesitancy and socio-demographics. The redcap data was relooked to fix any incorrect data or missed areas. Quantitative survey data was analyzed using descriptive statistics (frequencies, percentages) using STATA version 15.

At the end of the telephonic survey, women were asked if they would be willing to be contacted to participate in longer follow-up in-depth interviews.  Later, the research associate reached out to those who agreed for a telephonic in-depth interviews. of the 27 called, 15 women agreed to give an interview. The interviews were conducted till the saturation in the data was achieved. In depth interviews that lasted from 35-70 minutes. In depth interviews were collected until data saturation was reached, which was determined by the study team conducting the interviews. Some surveys and interviews were taken on multiple calls due to the time constraints of the participants. The interviews were conducted in Hindi. They were later transcribed and translated into English. A codebook based on the key themes covered in the interview was made to analyse the interviews. These were then analysed by the three members from the research team using dedoose software. In the context of covid-19 vaccine, key themes covered in the interview included general impact of covid-19 pandemic, its impact on their pregnancy, their own thoughts on covid-19 vaccine, thoughts of their family members, views of community and perinatal care provider on covid-19 vaccine, decision making, sources of information and understanding of bot technology.

Findings:
- To what extent did the 27% who did not want the vaccination overlap with the 30% who worried about the safety of the mother/baby?

Great question, we have added the following “Only 6% of women who were vaccinated reported this concern, while 26% of women who were not vaccinated did.”

- What were the main concerns of the remaining 70% of mothers?

*We have added the following:*

*Vaccines safety:* Most (95%) thought that the vaccine was effective and safe for the mother (93%) and baby (89%) (Table 2). Concerns related to health of the mother and baby were common in the survey data; for example, 22% were worried about safety for their baby in one way or another. Most (65%) had no concerns, 13% some other concerns (family members concern, general lock of trust, confusion, general “safety”); of note, none reported concerns regarding the mother’s health. Only 6% of women who were vaccinated reported this concern, while 26% of women who were not vaccinated did. In our qualitative research, women described their own, or their family member’s worry about the vaccine spoiling breast-milk quality and quantity, and the potential for impact on the physical and mental development of child.

- One of the 'main reasons' for lack of vaccination was the unwillingness of healthcare providers - what % of women reported this? (I assume 75%, based on line 156, but am uncertain)

*We have added the following:*

*Refusal on the part of health care providers:* One of the next more common reasons reported that women who wanted the vaccine had not gotten it was that community health workers and doctors were refusing to administer the vaccine to pregnant women; 13% of women who were not vaccinated reported this as the reason why. We did not specifically provide this response option in our questionnaire since we were not anticipating this situation; thus, we hypothesize this could have been the case for women more because we only have data on the women who selected “other” as a response option and told the interviewer that this was their reason. For example, women responded to the “other” option with statements like “I wanted to get it but dispensary staff and Nurse denied to vaccinate me. What should I do?” and “This vaccine is very effective & safe. Everyone should take this, but my doctor forbade me to take the vaccine due to my pregnancy.” This theme came up repeatedly in the qualitative interviews, supporting our hypothesis that this was even more common than survey data suggest.

- With regard to trust, in line 144 it states that 90% of women trusted govt/public health agencies, and in line 155 it states that 90% trusted perinatal care providers - do these refer to the same statistic?

These are two different statistics, hopefully the table clarifies this.

- Is there any data to back up the assertions about women's lack of mobility and empowerment?- Lines 173-176 seem out of place, perhaps move to the end of the findings?

We have expanded and shifted this section.

- Please be a bit clear about which findings are from the survey and which from the interviews.

We have tried to clarify throughout.

- If possible, accompany quotes with some basic information about the woman (age, pregnancy/post-partum/nursing status)

Thank you, we have added these.

- Fascinating finding - that trusted sources are at odds with sources women actually receive information from
We thought so too!

Discussion: 
- Line 209: please clarify 'larger proportion'

We have edited this to read “Healthcare provider refusal to vaccinate was a primary contributor to women in our sample being unable to receive a COVID-19 vaccine.”

- Although women's lack of mobility and empowerment is highlighted in the findings, it finds no place in the discussion (harder to address in an intervention, of course, but should be nevertheless be noted)

We have added the following:

“Second, we recommend the development of widespread public health education campaigns about the COVID-19 vaccine that specifically target women to ensure they are educated about COVID-19 vaccine safety, with a focus on safety in pregnancy and for the infant. Women in this setting often face restrictions on their mobility or empowerment, and thus educating women alone is insufficient—messaging must also target and engage family members both to be supportive of COVID-19 vaccination in pregnancy, but also more broadly to address gender equality.”

“To reach the underserved and high risk population of pregnant and breastfeeding women, it is imperative that India’s COVID-19 vaccine outreach simultaneously target households (23), female health care workers who are frontline along with other health care providers, and address social norms to increase vaccine uptake. Considering the complex interplay between trust in the government and health care providers, gender and social dynamics and norms, and other factors aside from supply are key to understanding and address vaccine hesitancy and vaccination—for COVID-10 and likely other vaccinations now and in the future, in India and globally.”

- Similarly, while suggestions focus on targeting women, the findings suggest working with family members as well

See above

Overall, some of the statements and assertions made in the paper require some evidence to back them up.

We have worked to ensure all statements are reflective of findings from our research.
