# Supplementary material for: The supply is there. So why can’t pregnant and breastfeeding women in rural India get the COVID-19 vaccine?
Source: PLOS Glob Public Health. 2022 Dec 6;2(12):e0001321. doi: 10.1371/journal.pgph.0001321 (PMC10022347; doi:10.1371/journal.pgph.0001321)
Supplement: Attachment — Submitted filename: RandR2_ReviewerResponse_24Oct2022.docx [file pgph.0001321.s003.docx]

October 24, 2022

To the editors and reviewers,

Thank you for your support and feedback on this paper, which we feel has improved greatly in this process. We have responded to each comment below, point by point. Please let us know if you need additional clarification,

Thank you

Nadia Diamond-Smith, on behalf of my co-authors

________________________________________________________________________________________________

Reviewer #2: Dear authors,

Your effort in addressing comments that were raised with respect to the original submission is noted and appreciated. There is more clarity in the methodology, detail in the findings, and analysis in the discussion - and these revisions significantly strengthen the paper. But more remains to be done, described below in order of importance.

Thank you for your continued support for this paper, we have addressed your comments below.

- In Table 2, is it possible to breakdown further between vaccinated and unvaccinated women? When not separated out, it is hard to identify specific drivers of vaccination/non-vaccination, and this is really the meat of the paper. Please distinguish between the two categories when describing your findings, and further reflect on them in the discussion.

Thank you for this suggestion. We have separated out the results in Table 2 by vaccinated and unvaccinated women.

- I understand the desire to protect participants, but by providing such little detail about the study site, a lot of context is lost. Would strongly suggest including some additional information.

We discussed as a team and decided we are comfortable now with sharing the states, we have added that most women were from Haryana, but some may have been in Punjab, UT Chandigarh, Uttar Pradesh, Uttarakhand or UT Delhi.

There are many other points require additional clarification:

- Lines 129-130: are phrased ambiguously - were all participants from 2 districts, or 60% of them?

We have clarified this to read “The WhatsApp groups through which we recruited (described below) was designed to serve a population residing in rural and suburban areas in two districts. However, since women migrate, our estimates are that about 60 % of the respondents were from these two districts in north India.”

- Line 131: rank in the middle of what?

We have added “, compared to other Indian districts,”

- Lines 129-134: were participants rural, or rural and suburban?

Apologies, removed “rural” since this was described above

- Lines 135-142: were participants up to 6 months post-partum, or up to 1 year post-partum?

Apologies for the lack of clarity, 1 year. Edited this throughout.

- Clarify how having or not having a ration card was a marker of poverty

We have added “Ration cards are provided based on the income level.” If the reviewer is interested in learning more, here is a link https://www.brookings.edu/blog/future-development/2022/04/14/raising-the-standard-time-for-a-higher-poverty-line-in-india/#:~:text=The%20time%20has%20come%20for,level%20some%2068%20percent%20higher

- Note the version of Dedoose used

Added that it was version 8.

- Line 188: XX XX - I assume details will be filled in?

Yes, will be filled in after review, since these are also authors.

- Line 192: what is bot technology, and what is its relevance to this study?

This was the formative work for an intervention using chatbots for vaccine knowledge for pregnant/breastfeeding women, so we asked these questions. We have removed the word “bot” so that its less confusing.

- Lines 209-211: This an important point and needs to be moved up further

In the introduction we say “Shortly before the Indian government issued guidance that the COVID-19 vaccine was approved for pregnant and breastfeeding women in India (July 2^nd^, 2021, Figure 1, before our study took place),” and also we have added this in the methods “All women in the WhatsApp group were connected to the government health care system, which provides free services.”

- Lines 211-213 - doesn't add up to 100% - what other categories included?

We have added “(the remaining were unsure).”

- Line 214+: Does this excerpt refer to one of the women in the 47%?

We did not connect the qualitative directly to the quantitative survey, although they were from the same sample.

- Lines 224-225 show high trust in vaccines, but line 226 onwards shows some concerns - are these not at odds?

Yes, feelings about vaccines and vaccine hesitancy are quite complex, this is one of the main points we are hoping readers will take away from our study—and it highlights the importance of mixed methods and asking more probing questions in both surveys and in depth interviews.

- Lines 244-245 - where is this stated in the hypothesis?

Thank you, you are right, this was not a pre-stated hypothesis. We have re-worded this to say “This theme came up repeatedly in the qualitative interviews, suggesting that this sentiment was even more common than survey data suggest.”

- Lines 274-275: "the health care system not wanting to vaccinate pregnant women" is awkward phrasing

Rewritten to read “Aside from reluctance among health care providers to vaccinate pregnant women,”

- Line 320: In Factors associated with being vaccinated, p values are not provided consistently, so it is hard to know whether the finding is significant or not

Where confidence intervals clearly do not cross 1, so do not provide p-values. We provide p-values for the places where the findings were almost significant at the 0.05 level, but Cis did cross 1. We only report on those findings that were significant or close to it by the definition described above.

- Lines 329-331 - is the association positive or negative?

Lower odds, as is noted at the end of the sentence.

- It is still not always clear what findings are from the phone surveys and/or the in-depth interviews

Apologies, we have gone through to try to make this clearer, but fundamentally, since these are exactly the same population, we feel that it is not critical to make this clear with each point. For the most part, we try to present numbers for the survey data and quotes accompanying points that came out of the qualitative.

- Table 1: the religion/Hindu row is not filled

Added for the survey (quantitative data), we did not ask this for the qualitative portion.

- The abstract is a little disorganized and I don't think it represents the content of paper effectively.

Agreed, we have edited and re-organized.

- Finally, the manuscript is filled with typos and grammatical errors: line 81 ("this is contrast"); line 105 ("vaccine uptake trust"); line 108 ("pregnancy women"); line 117 ("more, mixed methods"); line 141 ('oof' instead of 'of'); line 150 ("were called to again to..."); etc. I stopped noting these after one point, but please proof-read the paper carefully.

Thank you and apologies, we have edited these and did find more too, which we have edited

I do believe these are minor revisions and can be addressed easily. This is an important paper and it deserves to be published, and I look forward to seeing it in print.

Regards.
